# Immunoregulatory and anti-inflammatory properties of *Crocus sativus* (Saffron) and its main active constituents: A review

**DOI:** 10.22038/ijbms.2019.34365.8158

**Published:** 2019-04

**Authors:** Majid Zeinali, Mohammad Reza Zirak, Seyed Abdolrahim Rezaee, Gholamreza Karimi, Hossein Hosseinzadeh

**Affiliations:** 1 Department of Pharmacodynamics and Toxicology, School of Pharmacy, Mashhad University of Medical Sciences, Mashhad, Islamic Republic of Iran; 2 Social Security Organization, Mashhad, Islamic Republic of Iran; 3 Inflammation and Inflammatory Diseases Research Center, Mashhad University of Medical Science, Mashhad, Islamic Republic of Iran; 4 Pharmaceutical Research Center, Pharmaceutical Technology Institute, Mashhad University of Medical Sciences, Mashhad, Iran

**Keywords:** Anti-inflammatory, * Crocus sativus*, Cytokines Immunomodulatory, Saffron

## Abstract

The medicinal uses of saffron, the dried stigmas of *Crocus sativus* L., have very long history in food coloring agent, and flavoring agent as well as traditional medicine for the treatment of several diseases. *Crocus sativus* is rich in carotenoids that affect immunity. This review summarizes the putative immunoregulatory effects of saffron and its active its derivatives including crocin, crocetin and safranal. In modern studies, its active constituents including protective effects, anti-inflammatory activities and molecular mechanisms of saffron on thimmune system have been demonstrated. Furthermore, the beneficial effects of saffron on inhibition of serum levels nuclear transcription factor κB (NF-κB) p65 unit, tumor necrosis factor alpha (TNF-α), interferon gamma (IFN-γ) and some interleukin (IL) such as IL-1β, IL-6, IL-12, IL-17A were reported. Furthermore, saffron has been known as the antagonist of NF-κB and the agonist of peroxisome proliferator-activated receptor gamma (PPAR-γ). In addition, saffron down-regulates the key pro-inflammatory enzymes such as myeloperoxidase (MPO), cyclooxygenase-2 (COX-2), inducible nitric oxide synthase (iNOS), phospholipase A2, and prostanoids.

This review summarizes the protective roles of *C. sativus* and its constituents against the pathogenesis of immune diseases and understanding a better management of these problems. Taken together, the main bioactive constituents of saffron may have health-promoting with important benefits in immune-related disorders. Finally, our study indicates that these bioactive constituents can affect both cellular and humoral immunity functions.

## Introduction

**Figure 1:**
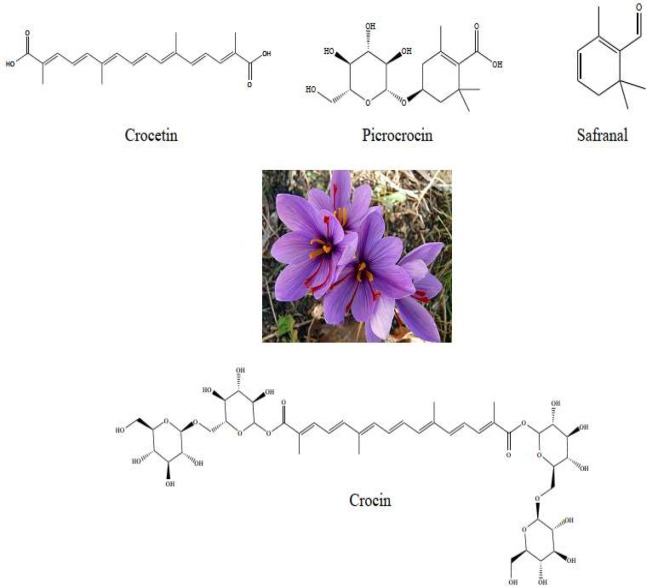
Saffron and its constitu ents

Saffron, the dried, dark red stigmas of *Crocus sativus *L. from Iridaceae family, is a well-known traditional herb that was mentioned in Iranian medical books (1, 2). This famous plant is now cultured largely in other places around the world such as Central Asia, Europe, India, Turkey and China (3, 4). The chemical components of saffron are sugars (63%), protein (12%), moisture (10%), fat (5%), minerals (5%) and 5% crude fiber (% w/w). More than 150 volatile compounds are present in saffron stigmas; the major bioactive compounds in this traditional herb are crocin (C_44_H_64_O_24_), picrocrocin (C_16_H_26_O_7_) and safranal (C_10_H_14_O) which are responsible for colors, taste and odor of saffron, respectively (Figure 1) (5). The chemical structure of crocin (mono- or di-glycosyl esters of crocetin) consists of crocetin as a central core and two sugars that are responsible for the color of the compound (6, 7). Interestingly, safranal is a monoterpene aldehyde, formed in saffron by hydrolysis from picrocrocin during drying and storage (8). Also, some several compounds such as mineral agents, anthocyanins, glycosides, alkaloids and some flavonoids including quercetin

 and kaempferol not only are presents in this plant but also presents in the saffron petal (9, 10). The main bioactive metabolites of the saffron spice are coming from the carotenoids (11, 12). Recently, several *in vitro* and *in vivo* modern studies have clearly documented that saffron has multiple putative biological activities, such as anti-cancer (13-15), anti-inflammatory (13, 16, 17), antioxidant, radical scavenging (18-21), antidepressant (22, 23), anti-allergic (24), anti-arthritic (25), anti-genotoxic (1), anti-aging (18), antihypertensive (26, 27), anti-angiogenesis (28-30), anti-atherogenic (31), antibacterial (32-34), anti-diabetic (35, 36), anti-obesity (37), neuroprotective (38-40), hepatoprotective (41-44), nephroprotective (45), cardioprotective (46, 47) and beneficial effects on reproductive system (48). 

Over the past few years, studies have revealed that inflammation and the immune system play a dominant role in the pathophysiology of some important pathological conditions like atherosclerosis, metabolic syndrome, cancer, neurodegenerative diseases, asthma and allergy. The extracts of *C. sativus* and its constituents have been tested as adjuvant treatment in mentioned disorders (49, 50). On the other hand, different *in vivo* and *in vitro* reports described immunoregulatory properties of saffron and its constituents (Table 1). Thereby, compounds with immunoregulatory properties may be effective for prevention and treatment such diseases. In this review, we focus on the new investigation about the immunomodulatory effect of saffron and its constitutes in recently studies.

***Safety evaluations of saffron and its main constituents***

Based on the new clinical studies, safety evaluation of saffron has been reviewed. The documents mentioned that saffron has demonstrated few well-tolerated side effects. The most frequent side effects of this medicinal plant were reported including dizziness, dry mouth, headache, fatigue, nausea, daytime drowsiness, constipation and sweating (22, 69-72). In several clinical trials, no side effects of this spicy food were observed (73-76) even in a dose of 100 mg/day (54). In a human trial, ingestion of crocin tablet (30 mg/day; 15 mg twice a day for 4 weeks) was associated with menometrorrhagia, dyspnea and agitation in three different patients (77). In another study with the same dose but 6 weeks ingestion, decreased appetite was reported in four patients (78). Ayatollahi *et al.* reported that oral administration of saffron tablets (200 or 400 mg/day, for 7 days) did not induce any signiﬁcant changes on plasma level of some coagulant and anticoagulant factors such as fibrinogen, factor VII, protein C and S, prothrombin time (PT) and partial thromboplastin time (PTT) in comparison with placebo group (79). In some cases, the ingestion of 2 g of saffron could cause gastrointestinal bleeding (80). Overall, up to dose 1.5 g/day of saffron is considered safe while doses equal or more than 5 g/day may have toxic effects and doses ≥ 20 g are fatal (81).

***Methodology (search strategy and selection criteria)***

A literature search was performed using searched SciVerse (Science Direct and Scopus), PubMed, SpringerLink, Wiley Online Library and Google Scholar databases to identify immunomodulatory effects of *C. sativus* (saffron) and its active constituents specially crocin, crocetin and safranal with experimental evidence of involvement in the immune system (last accessed on Jully 2018). The following keywords were used: ‘‘*Crocus sativus*’’, ‘‘saffron, ‘‘crocin’’, ‘‘crocetin’’, ‘‘picrocrocin’’, ‘safranal’’, ‘‘cytokines’’, ‘‘innate immunity’’, ‘‘adaptive immunity’’, and ‘immune system’’.

***A summary on immunomodulatory properties of saffron***

**Figure 2:**
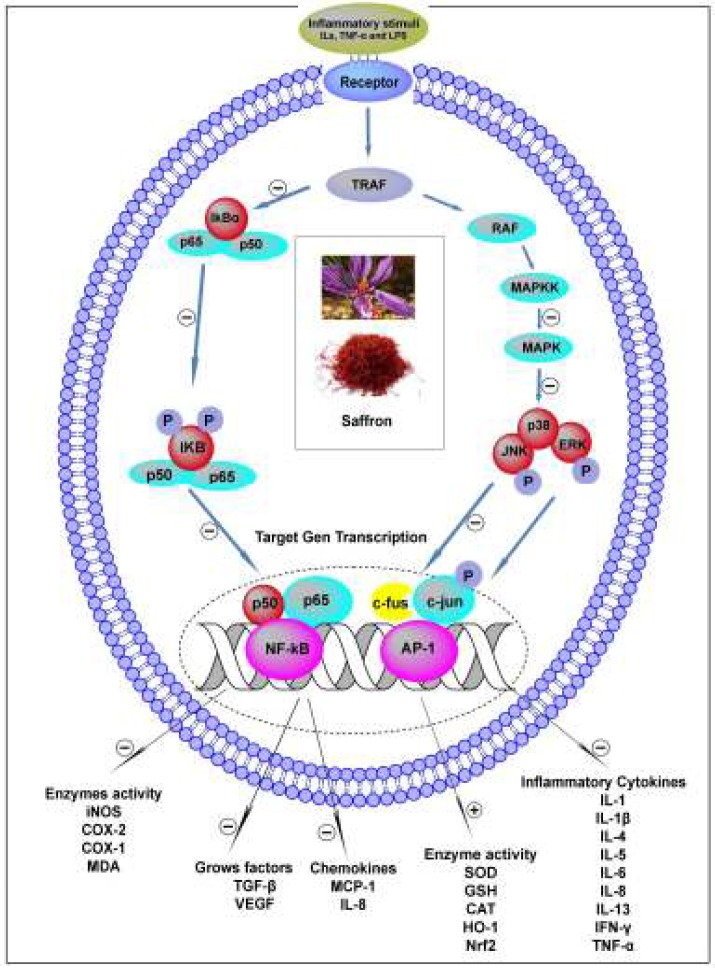
Immunomodulatory effects of saffron: transcription factors and signaling pathways

The anti-inflammatory potential of saffron is surely related to its strong antioxidant and radical scavenging virtues, which seems to chiefly ascribe to crocetin and crocins. Also, a variety of useful pharmacological activities of saffron stem from its ability to interact with various biological targets and different signaling pathways. Some studies suggested that the immunomodulatory activity of saffron may involve direct targeting of Toll-like receptors (TLRs), attributed to the regulation of various transcription factors such as nuclear factor (NF-κB), activator protein 1 (AP-1) and also their downstream signaling pathways (Figure 2). TLRs play a crucial role in the innate immune system by triggering pro-inflammatory signaling pathways in response to either external or internal stimuli (82). Moreover, NF-κB acts a vital role in producing pro-inflammatory cytokines such as IL-1, IL-2 and IFN-γ in T lymphocytes (83). Pradere *et al.* showed saffron has an inhibitory effect on producing pro-inflammatory cytokines like IL-1 production by suppressing NF-κB activity via the inhibition of I kappa B kinase-a (IKK-a) phosphorylation and prevention of nuclear translocation of the NF-κB p65 subunit (84).

***Biological activities of saffron on immuno-inflammatory cells***

*Anti-inflammatory activity on neutrophils*

*Saffron has been suggested as therapeutic herbal agents to avoid damages induced by neutrophil cells as the central cells in acute inflammatory processes. Within inflammatory processes, it is observed an increase in the number, mobility, lifespan, tissue influx ability and phagocytic activity of neutrophil cells (* 85 *). A toxicological study has demonstrated that subacute exposure to safranal (0.1, 0.5 and 1 ml/kg i.p for 3 weeks) did not have any significant changes on mice blood cellularity (neutrophils, lymphocytes, monocytes) and total white blood cells (WBCs) count (* 51 *). Also reported that saffron (100 mg daily for 6 weeks) did not have any significant effects on the count of WBCs and percentages of neutrophils, eosinophils and lymphocyte cells but the percentages of basophils in the saffron group were decreased significantly (* 54 *). Also, Tamaddonfard et al.* investigated the anti-inflammatory activity of crocins (25, 50, and 100 mg/kg) and safranal (0.5, 1, and 2 mg/kg) by decreasing the number of neutrophils count, infiltration of neutrophils in paw tissues and inflammatory pain responses in an animal model study (86). Accordingly, safranal (0.1, 0.5 and 1 ml/kg IP for 3 weeks) and saffron (100 mg daily for 6 weeks) did not have any significant effects on the count of WBC. Although, crocin (25, 50, and 100 mg/kg) and safranal (0.5, 1, and 2 mg/kg) could decrease immune cells in paw tissues of animals**.**

*Effect on natural killer cells cytotoxicity*

*There is a growing interest in the anti-cancer activity of saffron carotenoids (crocin and crocetin) on the modulation of immune responses by affecting the natural killer cell (NK-cell) activity in the elderly. The saffron carotenoids could increase NK-cell activity in the elderly (* 15 *).*

*Immunomodulatory effect on lymphoid and myeloid cells*

*Bayrami and coworker previously reported the effect of safranal (4, 8 and 16 *μg/ml in drinking water) and extract of saffron (0.1, 0.2 and 0.4 mg/ml) on total and differential count of WBC in ovalbumin (OVA)-sensitized guinea-pigs. The results illustrated that the administration of all concentrations of saffron aqueous extract and safranal significantly improved most types of WBCs but total WBCs number was only decreased in treated group with high concentration of the extract. Based on the results, it was concluded that safranal was more effective in the improvement of lymphocyte and eosinophil compared to the saffron extracts. However, the preventive effect of saffron extract on the total WBC count was more prominent than that of the safranal extract (53). In another study, it was observed that the hydroalcoholic extract of saffron (50, 10, and 200 mg/kg) reduced total WBCs count and decreased the percentage of eosinophils and neutrophils in lung lavage fluid of OVA-sensitized rats (87). In a similar study, the pretreatment of OVA-sensitized male Wistar rats with hydro-ethanolic extract of saffron reduced total WBC count, total red blood cells, total platelet count and decreased percentages of eosinophil and neutrophil in whole blood of animals (87). Consistent with these findings, a randomized double-blind placebo-controlled clinical trial study was done by Kianbakht and coworker. Based on their results, the effects of the sub-chronic daily use of saffron (100 mg for 6 weeks) showed some alterations in the immunological and hematological indices. Also, saffron has temporary immunomodulatory activities without any adverse effects. Furthermore, saffron did not have any significant effects on the WBCs count, the percentages of neutrophils, eosinophils and lymphocytes but the percentages of basophils in the saffron group were decreased significantly (54).

**Table 1:** Immunoregulatory effects of saffron and its active constituents

**Part of immune system**	**Subject/model**	**Constituents**	**Concentration/Dose, route and duration**	**Effects**	**References**
Neutrophils	Balb/c mice	Safranal	0.1, 0.5, or 1 mg/kg, i.p for 3 weeks	No signiﬁcant change in spleen/blood cellularity, HA, DTH, proliferation response to PHA, INF-γ/IL-4 ratios, INF-γ and IL-4 production. No adverse effect on spleen and bone marrow	(69)
	Wistar rats	Saffron petal extract	75, 150, 225, and 450 mg/kg i.p, for 14 days	No significant difference on neutrophils count	(104)
	Dunkin-Hartley guinea pigs	Hydro-ethanolic extract of *C.sativus*	0.1,0.2 and 0.4 mg/ml extract in drinking water	⬆ percentage of neutrophil	(71)
	Dunkin-Hartley guinea pigs	Safranal	4, 8 and 16 μg/ml in drinking water	⬆ percentage of neutrophil	
	Human	Saffron tablets (100 mg)	100 mg/day p.o for 6 weeks	No significant effect on neutrophils percentage	(59)
Macrophages	Mice	Crocin	0.5,1 and 1.5 mg/kg , gavage, 5 days	⬆ Macrophage activity ⬆ In vivo yeast phagocytic ability by peritoneal macrophages	(105)
	Mouse model of atherosclerosis (*ApoE−/−*mice)	Saffron aqueous extract	30,60,90 mg/kg/day for 4 weeks, gavage	⬇ Content of macrophages ⬆ Vascular Smooth Muscle Cells (SMC) ⬆ Plaque stability ⬇ MMP-3, MMP-9, MCP-1, IL-6 and TNF-α release Within the atherosclerotic plaques	(35)
	Wistar rats	Crocin	100 mg/kg/day) for 4 weeks, gavage	⬆ M2 macrophage polarization ⬇ Levels of pro-inflammatory cytokines such as IL-6, iNOS and TNF-α ⬆ Expression of anti-inflammatory cytokines (IL-4, IL-10 and TGF-β) ⬇ Expression and nuclear translocation of NF-κB p65 ⬇ Inflammatory response in a vitamin D3-induced rat coronary atherosclerosis model	(106)
Allergic inflammation	Murine model of chronic asthma (BALB/c mice)	Crocin	100 mg/kg intragastrically for 34 days	⬇ Ovalbumin-induced allergic asthma ⬇ The infiltration of the inflammatory cells in the airway ⬇ Total number of inflammatory cells in the bronchoalveolar lavage fluid (BALF) ⬇ The level of lung eosinophil peroxidase and serum OVA-specific IgE ⬇ Expression of lung eotaxin, p-ERK, p-JNK and p-p38 ⬇ IL-4,IL-8 IL-13 and IFN-γ in BALF	(24)
	Murine model of chronic asthma (Swiss Albino mice)	Crocin	25mg/kg/day orally for 16 days	⬇ Ovalbumin-induced allergic asthma ⬇ Inflammatory cell counts in BALF, lung total protein content and pulmonary edema ⬇ TNF-α, IL-4, and IL-13 ⬇ Serum lactate dehydrogenase (LDH) activity and lung malondialdehyde (MDA) content ⬆ Superoxide dismutase (SOD) activity, reduced glutathione (GSH) levels and serum and lung catalase activities	(107)
	Normal human bronchial epithelial cells (NHBE)	Safranal	10 and 100 ng/ml	⬇ Cytochrome c release and epithelial cell apoptosis ⬇ iNOS levels and NO production ⬇ Mitochondrial ROS production ⬇ Peroxy nitrite ionformation	(108)
		Crocin	10 , 100 and 1000 ng/ml	⬇ iNOS levels No significant effect on NO levels	
	Mouse model of asthma	Safranal	1 and 10 mg/kg For 8 days, from Day 20 to 27 once a day	⬇ Airway cellular infiltration ⬇ Inflammationscore, epithelial cell thickening , bronchial contraction and mucus hypersecretion ⬇ iNOS and NO level ⬇ Inflammatory cytokines (IL-5 and IL-13)	
Cytokine secretion	Human lymphocytes	*C. sativus* Extract	500 µg/mL	⬇ Secretion of IFN-γ and IL-10 in PHA stimulated cells ⬆ IFN-γ and IL-4 secretion in non-stimulated cells Inhibitory effect on Th2 cells and stimulatory effect on Th1 cells	(109)
	Pperipheral blood mononuclear cells (PBMC)	Safranal	0.1, 0.5 and 1 mM	⬇ IFN-γ and IL-10 secretion in PHA stimulated cells ⬆ IFN-γ secretion in non-stimulated cells ⬆ IFN-γ/IL-4 ratio No inhibitory effect of on IL-4 secretion Anti-inflammatory effects via stimulation Th1 and/or suppression Th2 lymphocyte subtype	(110)
	PC-12 cells	Crocin	1 and 10 µM	⬇ TNF-α levels and caspase 3 ⬆ Bcl-X_L_ and Bcl-2 ⬇ Cytosolic cytochrome c levels ⬇ TNF-α induced PC-12 death	(111)
	D-galactose-induced aging in mice	Crocin	10, 20 and 40 mg/kg for 42 days, i.p.	⬇ TNF-α and IL-6 in serum ⬇Malondialdehyde (MDA) as a lipid peroxidation marker ⬆ Glutathione content (GSH) ⬇ ALT, AST ⬆ Sex hormones (Testosterone and DHEA-SO_4_)	(112)
	Human with metabolic Syndrome	Saffron	100 mg/day for 12 weeks, p.o.	⬇ Total-cholesterol, LDL TG, FBS and hsCRP ⬆ HDL ⬇ Serum concentration of pro-inflammatory cytokines	(113)
	Streptozotocin (STZ)-induced diabetic rats	Saffron aqueous extract	10,20 and 40 mg/kg/day for 25 days, i.p.	⬇ Blood glucose, total lipids, triglycerides, total cholesterol and LDL-C ⬆ Serum HDL-C level ⬇ MDA levels and serum NO levels ⬆ GSH, SOD and CAT activities ⬇ Inflammatory cytokines such asTNF-α and IL-6)	(114)
	Rat model of arthritis	Crocin	10 and 20 mg/kg from 11th day up to 25th day, p.o.	⬇ MMP-13, MMP-3 and MMP-9 and HAases ⬇ TNF-α, IL-1β, NF-κB, IL-6, COX-2, PGE_2_ and ROS ⬆ GSH, SOD, CAT and GST	(80)
	Rabbit osteoarthritic model	Crocin	0.3 ml of 5 and 100 μM, intra-articular injection once per week for 5 weeks	⬇ Cartilage degeneration during Osteoarthritis progression ⬇ Gene expression of MMP-1, -3 and -13	(115)
	Chondrocytes	Crocin	5,25,50 and 100 µM	⬇ Gene and protein expression of MMP-1, -3 and -13 ⬇ NF-κB activity	
Neuropathy and Neuro-inflammation	Mouse model with neuropathic pain (CD1 mice)	Crocetin	5-50 mg/kg/day , intra subarachnoid space, for 12 days	⬇ Mechanical and thermal allodynia in spared nerve injury (SNI) mice ⬇ The production of IL-1β and TNF-α ⬇ Oxidative stress and ⬆ mitochondrial SOD activity	(116)
	Sciatic nerve injury in rats	Safranal	0.2 and 0.8 mg/kg/day for 10 days, i.p.	⬇ Cold and mechanical allodynia ⬇ MDA level	(117)

***Biological activities of saffron on inflammation and cytokines***

**Immunomodulatory effects on major pro-inflammatory cytokines**

Research projects clearly showed that saffron could decrease the pro-inflammatory responses (88-90). For example, the administration of saffron and ethanolic aqueous extracts could alleviate neuropathic pain in the chronic constriction injury model through the reduction of pro-inflammatory factors (IL-1β, IL-6 and TNF-α) in rats (88). Another study showed that crocin treatment (10, 20, 30 mg/kg/day, IP for 4 weeks) has a protective effect not only on kidney organs through reducing the oxidative stress in aged rats but also significantly reduced pro-inflammatory cytokines (TNF-α, IL-6 and IL-1β) in the renal tissue and serum (89). Also, safranal (100 mg/kg) decreased the expression of the inflammatory cytokines TNF-α, IL-1β, and mitogen-activated protein kinases (MAPKs), such as the p38 in spinal cord injury models, but elevated the expression of the IL-10 level after spinal cord injury. Also, results showed that safranal could suppress the expression of aquaporin-4 (AQP-4), which is related to spinal-cord edema (Figure 2). This study suggested that safranal could ameliorate neuronal function following spinal cord injury in rats (91). Consistent with these findings, in 2010, Nam *et al.* examined a new study whether crocin or crocetin can repress microglial activation in rat brain microglial cells. Their results suggested neuroprotective effects of crocin or crocetin by a decrease in the production of pro-inflammatory cytokines (IL-1β and TNF-α) in cultured rat brain microglia and inhibition of LPS-induced apoptosis in organotypic hippocampal slice cultures (90). So, they reported that both of them effectively reduced LPS-elicited NF-κB activation and crocin reduced the NO release from microglia which stimulated with amyloid-beta and INF-γ agents (90). Also, a new original study revealed that crocin (30 mg/kg) treated orally for 28 consecutive days was able to improve learning and memory of tramadol-treated rats and also decreased the neurotoxicity effects of tramadol on dark neurons and apoptotic cells in the hippocampus (92).

Furthermore, a study in mouse colon carcinogenesis model showed the inhibitory effects of crocin against inflammation which was associated with mouse and chemically induced colitis by azoxymethane and dextran sodium sulfate (DSS) in male ICR mice. After usage of crocins, the results confirm that crocins could improve colitis and colitis-related colon carcinogenesis induced chemically in animal by reducing mRNA expression of some pro-inflammatory cytokines and inducible inflammatory enzymes including IL-1β, IL-6, TNF-α, INF-γ, NF-κB, iNOS and COX-2 synthase in the colorectal mucosa and increasing in the nuclear factor erythroid 2–related factor 2 (Nrf2) mRNA expression of the mice that received DSS (93). In another study, Zhou *et al.* revealed that treatment of rats by crocetin may protect animal against burn-induced small intestinal injury by anti-inflammatory and antioxidant effects. Data showed that crocetin not only inhibited neutrophil accumulation in the small intestine but also reduced some pro-inflammatory response (IL-6, TNF-α) and NF-κB activation in burn model study (94). It has been shown that some pro-inflammatory cytokines (IL-1β, IL-6, and TNF-α) and inflammatory mediators (prostaglandin E-2 productions (PGE-2) and COX-2) were inhibited by crocin (20 mg/kg. IP) in Wistar rats model of arthritis (Figure 2) (65). Also, intraperitoneally pretreatment with saffron hydro-ethanolic extracts in all doses (5 mg/kg, 10 mg/kg, or 20 mg/kg) significantly reduced the TNF-α and ICAM-1 in a dose-dependent manner in ischemia/reperfusion-induced acute kidney injury in rats (11). A new original study revealed that crocin (30 mg/kg) treated orally for 28 consecutive days was able to improve learning and memory of tramadol-treated rats and also decreased DNs and apoptotic cells in the hippocampus.

Immunomodulatory effects on anti-inflammatory cytokines

Among the anti-inflammatory cytokines, some of them such as IL-1Ra, IL-4, IL-10 and TGF-β are more important in the immune system (82). There are several studies, which confirm the anti-inflammatory effects of saffron and its components in cytokine pathways (13, 95). 

Results clearly showed that safranal ameliorated serum levels of histamine in sensitized guinea-pigs (20) and safranal (0.025, 0.05, and 0.1 ml/kg) demonstrated protective effects against the sub-acute diazinon-induced immune toxicity (96). Moreover, Boskabady and coworkers previously reported the prophylactic effect of safranal (4, 8 and 16 μg/ml in drinking water) on tracheal responses of OVA-sensitized guinea-pigs as a model of asthma. The authors found that safranal (4, 8 and 16 µg/ml) could significantly decrease the IL-4, total NO and nitrite but increased the IFN-γ concentration, in the serum of animal in all concentrations (97). Another study reported that subacute exposure to safranal (0.1, 0.5 and 1 ml/kg IP for 3 weeks) did not have any significant changes on IFN-γ and IL-4 produced by isolated mice splenocytes (51).

***Immunomodulatory effects of saffron on cell signaling pathways***

*Immunomodulatory effects on MAPK pathway*

*MAPK members (such as the p38 and the c-Jun N-terminal kinase (JNK)) and NF-*κB signaling pathways are known as two important molecular targets for the development of potential inflammatory and anti-inflammatory factors. They modulate the transcription of many genes involved in the inflammation and inflammatory process in immune system (82). Increasing MAPKs activity and their involvement in the regulation of the synthesis of inflammation mediators, make them potential targets for anti-inflammatory therapeutic agents (98). There are studies showing that saffron and its major bioactive components regulated MAPKs pathway signaling.

For example, in an *in vivo* study, crocin inhibited the LPS-induced overexpression pro-inflammatory factors (such as IL-1β, TNF-α, IL-6) and iNOS and TLR-2 in rat intervertebral discs. Notably, crocin suppressed the LPS-induced activation of the MAPK pathway by inhibiting the phosphorylation of JNK. Also, it has shown that crocin could exert anti-inflammatory effects by suppressing the activation of JNK (16). In the same way, Xiong *et al.* investigated the effect of crocin on the pathway of MAPK cascade. Analysis of the variables after treatment proved that crocin significantly inhibited the level of phosphorylated MAPK (p-ERK, p-JNK, p38 and p-p38 protein) in lung tissues of OVA-challenged mice (Figure 2) (24). Recently, the protective effects of crocin on sub-acute bisphenol A-induced liver toxicity in rats through inhibition of oxidative stress and downregulation of MAPK and MAPK-activated protein kinases (MAPKAPKs) signaling pathways was reported. After administration of crocin 20 mg/kg, the results confirm that crocin could improve liver injury in an animal study and lowering the phosphorylation of ERK1, ERK2, JNK, MAPKAPK and subsequently their activities in animal (99). The protective effects of saffron extracts against doxorubicin-induced acute cardiotoxicity in isolated rabbit hearts submitted to 30 minutes global ischemia followed by 40 minutes reperfusion were reported. After administration of saffron extracts during the first minutes of reperfusion, the oxidative myocardial damages reduced significantly. The findings of this study proved that saffron inhibited the p38 MAPK pathway, and activated the AKT/mTOR/4EBP1 pathway in reperfusion and DOX-treated rabbit heart homogenates (100). In addition, the effects of crocetin (20 mg/kg, PO) on ischemia/reperfusion-induced retinal damage in mice showed that this major active compound of saffron could reduce the phosphorylation levels of NF-κB, p38, JNK, and c-Jun but not that of ERK 1/2 activation in the retina after ischemia/reperfusion (101). Recently a study indicated that saffron strongly enhanced glucose uptake and the phosphorylation of AMP-activated protein kinase/acetyl-CoA carboxylase and MAPKs, but not phosphatidylinositol 3-kinase/Akt (102). Also, a study reported that crocetin inhibited inflammation by blocking NF-κB signaling and diminished cardiac hypertrophy by blocking the reactive oxygen species (ROS)-dependent MAPK/ MEK/ERK1/2 pathway and GATA binding protein-4 (GATA-4) activation (103). 

Immunoregulatory effects on the NF-*κB pathway and target genes*

*NF-*κB activation acts as a crucial role in producing pro-inflammatory cytokines (such as IL-1, IL-2 and IFN-γ) in immune cells (83). Also, NF-κB regulates the expression of several important genes, such as COX-2, iNOS, TNF-α and cell surface adhesion molecules, which are involved in tumor initiation, promotion, and metastasis (93). There are several studies indicating that saffron and its constituents have an important role in inhibition the NF-κB and subunits (89-91). For example, it was observed that crocin has shown anti-inflammatory effects via inhibition the NF-κB, p50, and p65 subunits in carrageenan-induced paw edema and xylene-induced ear edema in rats (105). Moreover, crocin not only induced an anti-inflammatory response but also inhibited iNOS expression and NO production via downregulation of NF-κB activity in LPS-stimulated RAW 264.7 macrophages (106). A study by Cai *et al.* reported that crocetin decreased levels of monocyte chemoattractant protein 1 (MCP-1), IL-1β and TNF-α mRNA and protein expression in cardiac hypertrophy model. Moreover, both *in vitro* and *in vivo* models, suggested that crocetin abrogated NF-κB activation by disrupting DNA-binding and transcriptional activity by blocking the phosphorylation and degradation of IκB and IKKβ activation (Figure 2) (103). Saffron was found to inhibit the NF-κB pathway in the human umbilical vein endothelial cell (HUVEC). In this *in vitro* study crocetin ameliorated cell cytotoxicity, suppressed MCP-1 and IL-8 expressions through blocking NF-κB p65 signaling transduction in LPS-induced inflammatory responses (107). Another study has shown that oral administration of crocetin (50 mg/kg for 8 days) reduced the levels of NO and neutrophil infiltration in the inflamed colon in experimental ulcerative colitis in mice. Crocetin also reduced the levels of NO associated with the favorable expression of TH1 and TH2 cytokines and iNOS along with the downregulation of NF-κB (108).

Immunoregulatory effects on the iNOS & COX-2 pathways

NO is an important molecular mediator of signaling processes in a variety of physiological processes. Under pathological conditions, the production of NO is mainly stimulated by activation of iNOS (109). However, it has been shown that high amounts of NO may be detrimental to cellular and extracellular components and low NO concentration may play a central role in physiologic processes (110). There is an interaction between iNOS signaling with the COX-2 signaling. These inducible enzymatic pathways are two major inflammatory mediators implicated in inflammation that produce mediators (prostaglandins and nitric oxide) to cause inflammation and tissue damage (111). An experimental study was carried out by Xu *et al.* to evaluate the immunoregulatory effects of crocin on NO and COX enzymes activity. Crocin showed a dual inhibitory activity against the COX-1, 2 enzymes. Pretreatment animal by crocin (PO) dose-dependently inhibited the carrageenan-induced paw edema in rats and xylene-induced ear edema. Also, crocin significantly inhibited the PGE-2 in LPS-challenged RAW 264.7 (105). Crocins not only induced the expression of heme oxygenase-1 (HO-1) which leads to an anti-inflammatory response but also inhibited iNOS expression and NO production via downregulation of NF-κB activity in Ca^2+^/calmodulin-dependent protein kinase 4-PI3K/Akt-Nrf2 signaling cascades in LPS-stimulated RAW 264.7 macrophages (116). Another study addressed that, crocetin and crocin were identified as potent NO inhibitor when tested on the macrophages (104). Saffron aqueous extracts (40 and 80 mg/kg) significantly decreased serum TNF-α and iNOS activity in hippocampus tissue of streptozotocin (STZ)-induced diabetic rats (112). The anti-inflammatory effect of crocin has been shown by Kunihiro *et al*. They reported that dietary feeding of crocin (100 ppm and 200 ppm for 4 weeks) significantly suppressed mRNA expression of COX-2, iNOS, NF-κB, TNF-α, IL-1β, and IL-6 in the colitis and colitis-associated colorectal carcinogenesis in male ICR mice (Figure 2) (93). Moreover, the cardioprotective potential effects of crocin (40 mg/kg) on NO synthase expression in post-ischemic isolated rat heart have been shown previously. This study suggested that the protective effect of crocin may possibly be related to regulating of eNOS and iNOS expressions (113). The potent *in vitro* and *in vivo* anti-inflammatory activity of crocetin was reported. Administration orally mice with crocetin in methylcholanthrene (MCA)-induced rodent tumor model showed the anti-inflammatory effect via downregulating IL-1β, TNF-α and polymorphonuclear cells (PMN). Further, crocetin decreased the COX-2 production in cervical cancer cells (Figure 2) (114).

Immunomodulatory effects on anti-allergic, asthma and immunoglobulin production

There are many pieces of evidence that inflammatory cells are involved in the pathogenesis of airway inflammation and asthma symptoms. Asthma is tightly related to the imbalance of Th1/Th2 cells pro-inflammatory as well as an increased of NO level (115). The Th1 cells primarily produce IL-2, IFN-γ and TNFα, whereas Th2 cells produce other cytokines, such as IL-4, IL-5, IL-6, IL-9, IL-10, and IL-13 (116). It is accepted that increased productions of some cytokines such as IL-4, IL-5 and IL-13 play vital roles in the inflammatory mechanism of allergic asthma. (117). A study addressed that crocin treatment significantly suppressed airway inflammation and IL-4, IL-5, IL-13 and tryptase, lung eosinophil peroxidase (EPO) activity in lung lavage fluid in a murine model of allergic airway disease. After treatment of crocin, it inhibited the MAPK pathway by affecting on p-JNK, p-ERK, and p-p38 expression in the allergic model in mice (24). Also, another study reported that saffron extract caused a reduction in IL-4 level in serum of sensitized animals while IFN-γ levels significantly increased. The levels of Th1/Th2 cytokines (IFN-γ/IL-4 ratio) played a vital role in the occurrence of airway inflammation and asthma. The increasing IFN-γ/IL-4 ratio in animals treated with saffron extract may indicate that this extract has stimulatory effects on Th1 and suppressive effects on Th2 cells (97). Also, the effect of safranal on tracheal responsiveness OVA-guinea pigs was examined recently. It showed that safranal (4, 8 and 16 μg/ml) significantly decreased IL-4 but increased IFN-γ levels in serum of animals. Moreover, the total NO and nitrite levels were significantly decreased in serum as well as increased Th1/Th2 balance in sensitized animals (97). The sub-chronic daily use of saffron (100 mg for 6 weeks) in a randomized double-blind placebo-controlled clinical trial study showed that saffron after 3 weeks could increase the IgG concentration but decreased the IgM concentration in comparision with the baseline and placebo groups (84).

## Conclusion

Numerous health problems can be treated by application of medicinal plants and their derivatives. The discovery and isolation of more specific immunoregulatory medicinal plants with anti-inflammatory effects, can improve our modern life quality. For this purpose, saffron (*C. sativus* L.) has been safely used in traditional medicine for a long period of time. Recently, the application of this traditional medicinal plant to the food and pharmaceutical industries is of great deal of interest. Based on the available evidence, immunomodulatory properties of saffron and its main bioactive compounds on the immune system are mediated by various mechanisms such as modulation of innate immunity and acquired immunity component. However, most of the pharmacological activities of saffron are related to the presence of crocin and crocetin. It appears that saffron can modulate MAPK and NF-κB pathways. Saffron controls the expression of genes encoding the pro-inflammatory cytokines (such as IL-1, IL-2, IL-6, TNF-α), inducible enzymes (e.g., COX-2 and iNOS), adhesion molecules (e. g., ICAM, VCAM, E-selectin), chemokines and some of the acute phase proteins, all of which play important roles in controlling most inflammatory processes in immune system. Accordingly, saffron and its components could be also considered as a promising immunoregulatory agent in the treatment of immune disorders.
